# Atherogenic Index of Plasma and Residual Risk in Anticoagulated Patients With Atrial Fibrillation: The Prospective Murcia Atrial Fibrillation Project III Cohort

**DOI:** 10.1161/JAHA.125.046694

**Published:** 2026-03-10

**Authors:** Eva Soler‐Espejo, Yang Chen, José M. Rivera‐Caravaca, María P. Ramos‐Bratos, Francisco Marín, Vanessa Roldán, Gregory Y. H. Lip

**Affiliations:** ^1^ Department of Hematology, Hospital Clínico Universitario Virgen de la Arrixaca Instituto Murciano de Investigación Biosanitaria, University of Murcia Murcia Spain; ^2^ Liverpool Centre of Cardiovascular Science at University of Liverpool Liverpool John Moores University and Liverpool Heart and Chest Hospital Liverpool UK; ^3^ Faculty of Nursing, Instituto Murciano de Investigación Biosanitaria CIBERCV, University of Murcia Murcia Spain; ^4^ Department of Cardiology, Hospital Clínico Universitario Virgen de la Arrixaca, Instituto Murciano de Investigación Biosanitaria CIBERCV, University of Murcia Murcia Spain; ^5^ Department of Clinical Medicine Aalborg University Aalborg Denmark; ^6^ Medical University of Bialystok Bialystok Poland

**Keywords:** atherogenic index of plasma, atrial fibrillation, major adverse cardiovascular event, residual risk, thromboembolism, Atrial Fibrillation

## Abstract

**Background:**

Patients with atrial fibrillation (AF) remain exposed to residual thromboembolic and cardiovascular risk despite oral anticoagulation. The Atherogenic Index of Plasma (AIP) reflects atherogenic burden, but its prognostic value in anticoagulated AF is uncertain.

**Methods:**

This prospective cohort study included consecutive outpatients with AF initiating oral anticoagulation between January 2016 and November 2021. AIP was calculated from baseline triglyceride and high‐density lipoprotein cholesterol levels. Patients were stratified into low and high AIP groups using an outcome‐driven cut‐off. Primary outcomes were thromboembolic events and major adverse cardiovascular events. Secondary outcomes included cardiovascular and all‐cause death. Associations were assessed using restricted cubic spline models and multivariable Cox regression analyses adjusted for AF‐related comorbidities and concomitant therapies.

**Results:**

Among 2535 patients (52.4% women; median age, 76 years [interquartile range, 69–82 years]) followed up for 1.81±0.50 years, thromboembolic events occurred in 187 (7.4%) and major adverse cardiovascular events in 254 (10.0%). Restricted cubic spline models showed significant nonlinear associations with thromboembolic events (overall *P*<0.001; nonlinear *P*=0.007) and major adverse cardiovascular events (overall *P*<0.001; nonlinear *P*=0.040). High AIP was independently associated with an increased risk of thromboembolic events after adjustment for conventional comorbidities (model 1: adjusted hazard ratio [aHR], 1.47 [95% CI, 1.07–2.02]), with the association remaining significant after further adjustment for commonly prescribed concomitant treatments (model 2: aHR, 1.38 [95% CI, 1.01–1.88]). Similar results were observed for major adverse cardiovascular events (model 1: aHR, 1.40 [95% CI, 1.07–1.84]; model 2: aHR, 1.35 [95% CI, 1.03–1.76]). No significant associations were found for mortality outcomes.

**Conclusions:**

Elevated AIP identifies anticoagulated patients with AF at increased residual thromboembolic and cardiovascular risk.

**Nonstandard Abbreviations and Acronyms**
aHR: adjusted hazard ratio
AIP: Atherogenic Index of Plasma
DOAC: direct‐acting oral anticoagulant
MACE: major adverse cardiovascular event
OAC: oral anticoagulant
VKA: vitamin K antagonist

**Clinical Perspective**

**What Is New?**

- Elevated Atherogenic Index of Plasma is independently associated with an increased risk of thromboembolic events and major adverse cardiovascular events in anticoagulated patients with atrial fibrillation, even after adjusting for conventional risk factors and concomitant treatments.

**What Are the Clinical Implications?**

- The Atherogenic Index of Plasma offers a complementary biomarker to conventional clinical risk scores (eg, CHA_2_DS_2_‐VASc [Congestive heart failure, Hypertension, Age ≥75 years (2 points), Diabetes, Stroke/transient ischemic attack/thromboembolism (2 points), Vascular disease, Age 65–74 years, Sex category]) for identifying high‐risk patients with atrial fibrillation with underlying cardiometabolic dysregulation, improving risk stratification beyond clinical and lipid measures alone.

Atrial fibrillation (AF) affects ≈1% to 2% of the general population, with an increasing prevalence, because of population aging and the rising prevalence of cardiovascular comorbidities.^1^ AF remains a major contributor to morbidity and mortality, given its association with ischemic stroke and other cardiovascular complications.^2^ Although oral anticoagulant (OAC) therapy significantly reduces the risk of thromboembolic events in patients with AF, a substantial residual risk persists.^3^

This residual risk highlights the clinical complexity and multifactorial nature of AF‐related complications,^4^ where various clinical and biological factors, including age, comorbidities, systemic inflammation, and atherogenic dysregulation, may continue to drive adverse outcomes despite adequate OAC treatment.^5^ These observations underscore the need for improved risk stratification strategies that extend beyond conventional clinical scoring systems, with the aim of identifying high‐risk individuals and informing more comprehensive preventive approaches.^6^

The Atherogenic Index of Plasma (AIP), derived from serum triglyceride and high‐density lipoprotein cholesterol (HDL‐C) levels, has emerged as a reliable marker of atherogenic risk.^7^ By capturing the balance between atherogenic and antiatherogenic lipoproteins, AIP offers a comprehensive reflection of the atherogenic potential of lipid profiles.^8^ Elevated AIP has been independently associated with adverse cardiovascular outcomes in diverse high‐risk populations, including individuals with diabetes,^9^, ^10^ coronary artery disease,^11^, ^12^ and cardiovascular‐kidney‐metabolic syndrome.^13^ Indeed, cumulative exposure to elevated AIP has been linked to an increased risk of ischemic stroke in the general population.^14^

Despite these findings, the prognostic utility of AIP in anticoagulated patients with AF remains poorly characterized. In this context, AIP may help identify a cardiometabolic high‐risk phenotype and serve as a complementary tool to existing clinical risk scores for refining residual thromboembolic risk assessment.

Accordingly, this study aimed to assess the association between AIP and adverse cardiovascular outcomes in a prospective cohort of patients with AF receiving OAC therapy.

## METHODS

The data used in this study were obtained from the Murcia AF Project III cohort and are not publicly available because of data protection and ethical restrictions. Derived data supporting the findings of this study are available from the corresponding author, Vanessa Roldán, on reasonable request.

### Study Design, Setting, and Participants

This prospective, observational, cohort study, the Murcia AF Project III, enrolled outpatients newly diagnosed with any type of AF who were naïve to OAC therapy and initiated treatment with either vitamin K antagonists (VKAs) or direct‐acting OACs (DOACs) at 2 anticoagulation clinics located in Murcia, Spain, between January 1, 2016, and November 30, 2021. During the study period, although DOACs were authorized for stroke prevention in nonvalvular AF, their de novo prescription in Spain was frequently subject to regulatory and reimbursement restrictions, which limited their initial use in routine clinical practice and contributed to continued VKA prescription.^15^

Eligible participants were adults aged ≥18 years. Patients with prosthetic heart valves, rheumatic mitral valve disease, or other significant valvular abnormalities were excluded. No additional exclusion criteria were applied. All patients meeting the inclusion criteria were consecutively enrolled, as this was an all‐comers study with no prior sample size calculation.

At baseline, comprehensive clinical data were collected, including sociodemographic characteristics, anthropometric measurements, comorbidities, concomitant pharmacologic therapies, and routine laboratory parameters. All baseline data were obtained from electronic health records and contemporaneous clinical assessment, based on documented physician diagnoses and standard clinical criteria. Stroke risk was assessed using the CHA_2_DS_2_‐VASc (Congestive heart failure, Hypertension, Age ≥75 years [2 points], Diabetes, Stroke/transient ischemic attack/thromboembolism [2 points], Vascular disease, Age 65–74 years, Sex category) and CHA_2_DS_2_‐VA (Congestive heart failure, Hypertension, Age ≥75 years [2 points], Diabetes, Stroke/transient ischemic attack/thromboembolism [2 points], Vascular disease, Age 65–74 years) scores,^16^, ^17^ whereas bleeding risk was evaluated using the HAS‐BLED (Hypertension, Abnormal renal/liver function, Stroke, Bleeding history or predisposition, Labile international normalized ratio, Elderly [>65 years], Drugs/alcohol concomitantly) score.^18^

The study protocol was approved by the Ethics Committees of University Hospital Morales Meseguer (reference: EST:20/16) and University Hospital Virgen de la Arrixaca (reference: 2020‐11‐12‐HCUVA). It was conducted in accordance with the ethical principles of the 1964 Declaration of Helsinki and its subsequent amendments. Written informed consent was obtained from all participants before inclusion.

### Estimation and Stratification of the AIP

At baseline, laboratory assessments were performed to determine serum triglyceride and HDL‐C levels. In accordance with previous studies,^19^, ^20^ AIP was calculated using the established formula: AIP =log_10_ (triglycerides [mmol/L]/HDL‐C [mmol/L]). Participants were stratified into low and high AIP groups based on a cutoff value derived from subsequent statistical analysis.

### Follow‐Up and Clinical Outcomes

Follow‐up was conducted through in‐person interviews during routine outpatient visits to the anticoagulation clinic, in alignment with standard clinical care. For patients who missed scheduled appointments, relevant information and vital status were obtained via review of medical records and follow‐up telephone calls. Under this structured follow‐up strategy, outcome and vital status information was available for all participants, and no patients had unknown follow‐up status. As an observational study, all follow‐up procedures were embedded within routine clinical practice, with no additional interventions or study‐specific visits implemented.

Clinical outcomes were ascertained through systematic review of electronic health records, including hospital discharge reports, imaging findings, and laboratory data, and were recorded according to the final clinical diagnosis documented by the treating physician. All outcome events were reviewed and confirmed by experienced clinicians at the participating centers.

The primary end points were: (1) the occurrence of thromboembolic events, defined as a composite of ischemic stroke, transient ischemic attack (TIA), or systemic embolism; and (2) major adverse cardiovascular events (MACE), comprising myocardial infarction, ischemic stroke/TIA, or cardiovascular death. Secondary end points included cardiovascular death and all‐cause death. Each adverse event was documented individually, and only the first occurrence of each event type was recorded. In time‐to‐event analyses, patients were censored at death (for nonfatal end points) or at the administrative end of follow‐up. The follow‐up period continued until patient death or a maximum of 2 years, whichever occurred first. Accordingly, censoring reflected administrative end of follow‐up or death rather than loss to follow‐up.

### Statistical Analysis

Continuous variables were expressed as mean±SD or median and interquartile range (IQR), as appropriate. Categorical variables were presented as absolute frequencies and percentages. Group comparisons were performed using Student *t*‐test or the Mann–Whitney *U* test for continuous variables, and Fisher exact test or Pearson χ^2^ test for categorical variables.

The cutoff value for AIP grouping was determined using the “survminer” package in R,^21^, ^22^, ^23^ based on maximally selected rank statistics to optimize separation of time‐to‐event outcomes.

To assess both overall and nonlinear associations between AIP and the primary outcomes, restricted cubic spline analyses were conducted with AIP as a continuous variable and adjusted for conventional cardiovascular risk factors and comorbidities (age, sex, AF type, hypertension, diabetes, prior stroke/TIA/thromboembolism, vascular disease, heart failure, chronic kidney disease, dyslipidemia, and smoking habit). The reference point for the splines was set at the identified cutoff. The number of knots was selected on the basis of the Akaike Information Criterion and Bayesian Information Criterion, with the final models using 3 knots placed at the 10th, 50th, and 90th percentiles of AIP.

Incidence rates with 95% Poisson CIs for the primary outcome were calculated for each AIP group. Rate differences were estimated using the low AIP group as the reference.

Kaplan–Meier survival curves were constructed to depict event‐free survival across AIP groups, with comparisons made using the log‐rank test.

Then, multivariable Cox proportional hazards models were used to assess the independent association between AIP and both primary and secondary outcomes, treating AIP as a categorical variable. The following models were applied: model 1, adjusted for classic cardiovascular risk factors and comorbidities (age, sex, AF type, hypertension, diabetes, prior stroke/TIA/thromboembolism, vascular disease, heart failure, chronic kidney disease, dyslipidemia, and smoking habit); and model 2, further adjusted for commonly prescribed concomitant treatments (OACs, lipid‐lowering agents, oral hypoglycemic agents, insulin, and antiplatelet therapy). Adjusted hazard ratios (aHRs) with corresponding 95% CIs were reported. The proportional hazards assumption was tested using Schoenfeld residuals, and no significant violations were observed.

Cumulative incidence functions were estimated for thromboembolic events and MACE using competing risk methods. For each outcome, the event of interest was defined as the first occurrence of the respective event, whereas death without a prior event was considered a competing risk. Patients without events were censored at the end of follow‐up. Cumulative incidence curves were constructed according to AIP categories and compared using the Gray test. Follow‐up time was calculated from baseline to the first occurrence of the event of interest, the competing event, or censoring, with a maximum follow‐up of 2 years.

Subgroup analyses were conducted to evaluate the consistency of the associations between AIP and both thromboembolic events and MACE across clinically relevant strata, including age, sex, AF type, CHA_2_DS_2_‐VASc risk category, hypertension, diabetes, history of stroke/TIA/thromboembolism, vascular disease, lipid‐lowering therapy, and type of OAC (DOACs versus VKAs). These analyses were performed after adjustment for conventional cardiovascular risk factors and comorbidities and were based on formal tests of interaction by including multiplicative interaction terms in the multivariable Cox regression models. As the primary objective of these analyses was to assess effect modification through interaction testing rather than to interpret within‐subgroup estimates independently, correction for multiple comparisons was not applied.

For thromboembolic events, receiver operating characteristic curve analyses were performed to evaluate the discriminative performance of the CHA_2_DS_2_‐VASc score modeled as a categorical variable reflecting clinically applied risk strata (low, intermediate, and high risk), AIP, and a combined model incorporating both variables. Areas under the receiver operating characteristic curve (AUCs) were estimated with corresponding 95% CIs.

To assess the incremental predictive value of AIP beyond categorical CHA_2_DS_2_‐VASc, reclassification analyses were conducted using absolute 2‐year risk thresholds of 1%, 5%, and 10%. Net reclassification improvement and integrated discrimination improvement were calculated on the basis of predicted risks derived from logistic regression models, comparing categorical CHA_2_DS_2_‐VASc alone with the combined categorical CHA_2_DS_2_‐VASc plus AIP model. Net reclassification improvement and integrated discrimination improvement estimates were obtained using the “PredictABEL” package in R.

All statistical tests were 2‐sided, and *P*<0.05 was considered statistically significant. Analyses were performed using Stata v18.0 (StataCorp LLC, College Station, TX), R (R Foundation for Statistical Computing, Vienna, Austria), and SPSS v25.0 (IBM Corp, Armonk, NY).

## RESULTS

The overall cohort was composed of 3259 patients with AF (52.8% women; median age, 77 years [IQR, 70–83 years]). Of these, 2535 patients (52.4% women; median age, 76 years [IQR, 69–82 years]) had complete lipid profiles, including triglycerides and HDL‐C measurements, and were included in the present analysis. The median CHA_2_DS_2_‐VASc and CHA_2_DS_2_‐VA scores were 4 (IQR, 3–5) and 3 (IQR, 2–5), respectively, and the median HAS‐BLED score was 3 (IQR, 2–4). Regarding OAC treatment, 1725 patients (68.1%) were receiving DOACs, whereas 810 patients (31.9%) were on VKAs (Table S1).

**Figure 1:**
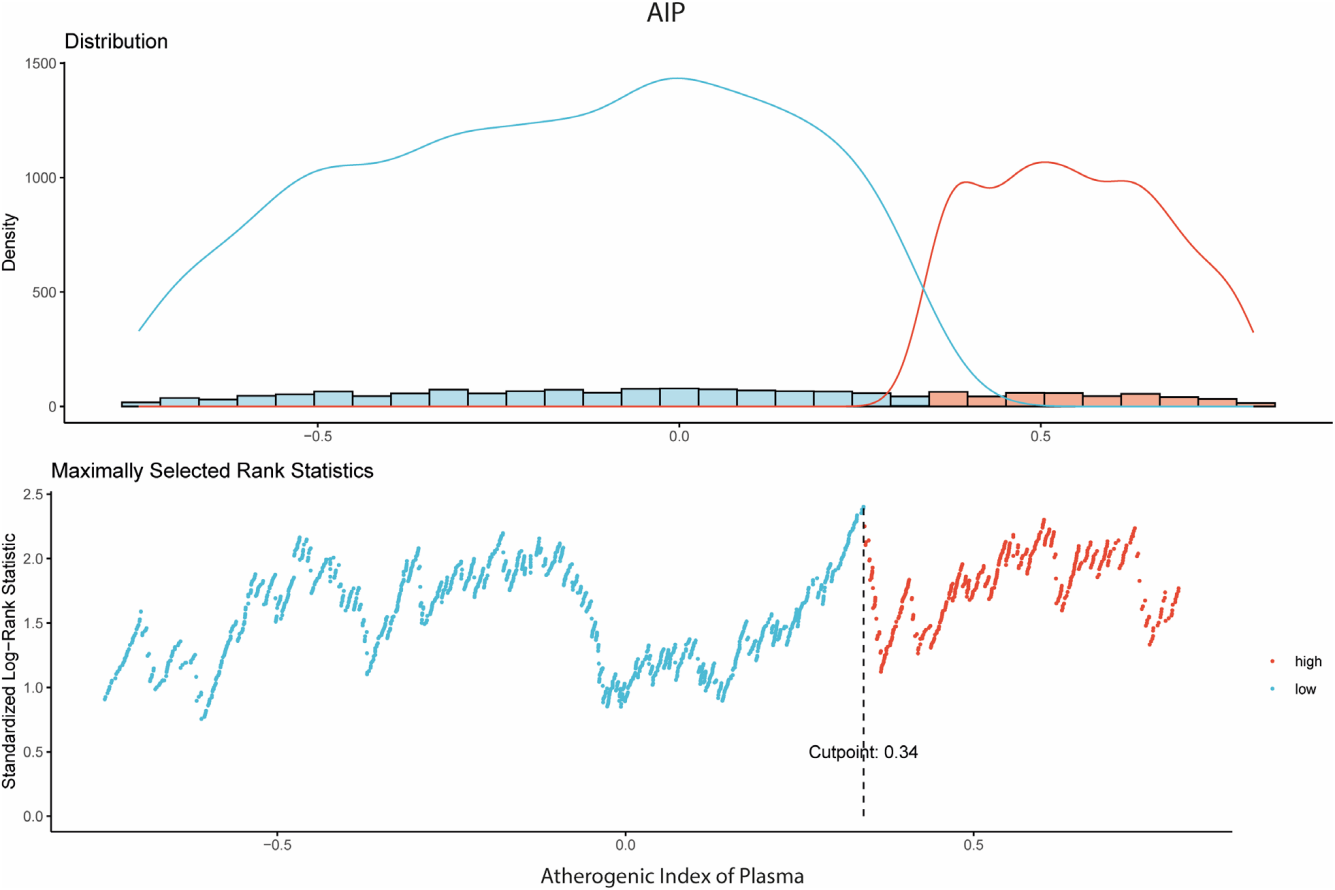
Visualization of the optimal cutoff point for thromboembolic events. AIP indicates Atherogenic Index of Plasma.

The median baseline triglyceride level was 1.26 mmol/L (IQR, 0.95–1.69 mmol/L), and the median HDL‐C level was 1.27 mmol/L (IQR, 1.03–1.55 mmol/L). On the basis of the identified AIP cutoff (Figure 1), 1793 patients (70.7%) were assigned to the low AIP group, and 742 patients (29.3%) were assigned to the high AIP group.

Baseline characteristics across AIP groups are shown in Table 1. Patients in the high AIP group had a greater comorbidity burden (eg, hypertension, diabetes, heart failure, vascular disease, renal impairment, dyslipidemia, and chronic obstructive pulmonary disease/obstructive sleep apnea) compared with those in the low AIP group.

**Table 1:** Baseline Clinical Characteristics According to AIP Group

Characteristic	Low AIP (N=1793)	High AIP (N=742)	*P* value
Demographics			
Age, median (IQR), y	77 (70–83)	75 (67–81)	<0.001
Female sex, n (%)	979 (54.6)	349 (47)	<0.001
AF type, n (%)			
Persistent	1156 (64.5)	455 (61.3)	0.146
Paroxysmal	637 (35.5)	287 (38.7)	
Comorbidities, n (%)			
Hypertension	1506 (84)	651 (87.7)	0.019
Diabetes	572 (31.9)	409 (55.1)	<0.001
Heart failure	340 (19)	179 (24.1)	0.004
History of stroke/TIA/thromboembolism	403 (22.5)	192 (25.9)	0.074
Vascular disease*	353 (19.7)	189 (25.5)	0.002
Renal impairment	351 (19.6)	196 (26.4)	<0.001
Dyslipidemia	985 (54.9)	520 (70.1)	<0.001
COPD/OSA	370 (20.6)	189 (25.5)	0.009
History of relevant bleeding	280 (15.6)	129 (17.4)	0.297
Liver disease	73 (4.1)	41 (5.5)	0.133
History of cancer	253 (14.1)	78 (10.5)	0.017
Smoking habit	412 (23)	201 (27.1)	0.032
Alcoholism	159 (8.9)	80 (10.8)	0.154
Concomitant treatment, n (%)			
Oral anticoagulants			
DOACs	1249 (69.7)	476 (64.2)	0.008
VKAs	544 (30.3)	266 (35.8)	
Antiarrhythmics	306 (17.1)	122 (16.4)	0.746
ACE inhibitors	437 (24.4)	195 (26.3)	0.337
ARBs	804 (44.8)	342 (46.1)	0.595
Calcium channel blockers	540 (30.1)	208 (28)	0.318
β‐Blockers	1162 (64.8)	540 (72.8)	<0.001
Diuretics	1030 (57.4)	446 (60.1)	0.223
Lipid‐lowering agents	969 (50.6)	420 (56.6)	0.007
Oral hypoglycemic agents	431 (24)	321 (43.3)	<0.001
Insulin	123 (6.9)	100 (13.5)	<0.001
Antiplatelet therapy	243 (13.6)	137 (18.5)	0.002
Analytical parameters, median (IQR), mmol/L			
Triglycerides	1.1 (0.9–1.3)	2 (1.7–2.5)	<0.001
HDL‐C	1.4 (1.2–1.7)	1 (0.9–1.2)	<0.001
Stroke and bleeding scores, median (IQR)			
CHA_2_DS_2_‐VA	3 (2–4)	4 (3–5)	<0.001
CHA_2_DS_2_‐VASc	4 (3–5)	4 (3–6)	0.011
HAS‐BLED	3 (2–4)	3 (2–4)	0.009

ACE indicates angiotensin‐converting enzyme; AF, atrial fibrillation; AIP, Atherogenic Index of Plasma; ARB, angiotensin II receptor blocker; CHA_2_DS_2_‐VA, Congestive heart failure, Hypertension, Age ≥75 years (2 points), Diabetes, Stroke/transient ischemic attack/thromboembolism (2 points), Vascular disease, Age 65–74 years; CHA_2_DS_2_‐VASc, Congestive heart failure, Hypertension, Age ≥75 years (2 points), Diabetes, Stroke/transient ischemic attack/thromboembolism (2 points), Vascular disease, Age 65–74 years, Sex category; COPD/OSA, chronic obstructive pulmonary disease/obstructive sleep apnea; DOAC, direct‐acting oral anticoagulant; HAS‐BLED, Hypertension, Abnormal renal/liver function, Stroke, Bleeding history or predisposition, Labile international normalized ratio, Elderly (>65 years), Drugs/alcohol concomitantly; HDL‐C, high‐density lipoprotein cholesterol; IQR, interquartile range; TIA, transient ischemic attack; and VKA, vitamin K antagonist.

* Vascular disease includes coronary artery disease and/or peripheral artery disease.

### Primary Outcomes Across AIP Groups

During a mean follow‐up of 1.81 years (SD, 0.5 years), 187 patients (7.4%) experienced a thromboembolic event, and 254 patients (10.0%) experienced a MACE.

As shown in Table 2, incidence rates for both thromboembolic events (3.68 versus 5.08 events per 100 person‐years; *P*=0.047) and MACE (5.05 versus 6.39 events per 100 person‐years; *P*=0.051) were lower in the low AIP group compared with the high AIP group, although the difference in MACE rates was of borderline statistical significance.

**Table 2:** Incidence Rate and RD for the Different Outcomes According to the AIP Group

Variable	Low AIP		High AIP		RD (95% CI)	*P* value
	No. (%)	Incidence rate (95% CI)*	No. (%)	Incidence rate (95% CI)*		
Any thromboembolic event	120 (6.7)	3.68 (3.05 to 4.39)	67 (9)	5.08 (3.93 to 6.45)	1.40 (0.02 to 2.78)	0.047
MACE	166 (9.3)	5.05 (4.31 to 5.88)	88 (11.9)	6.62 (5.31 to 8.16)	1.57 (−0.01 to 3.15)	0.051
Cardiovascular death	73 (4.1)	2.16 (1.69 to 2.71)	36 (4.9)	2.59 (1.81 to 3.58)	0.43 (−0.55 to 1.41)	0.392
All‐cause death	193 (10.8)	5.71 (4.93 to 6.57)	89 (12)	6.39 (5.14 to 7.87)	0.69 (−0.87 to 2.24)	0.386

AIP indicates Atherogenic Index of Plasma; MACE, major adverse cardiovascular events; and RD, rate difference.

* Per 100 person‐years.

### Secondary Outcomes Across AIP Groups

During the follow‐up period, 109 patients (4.3%) died from cardiovascular causes, and 282 patients (11.1%) died from any cause.

Cardiovascular death (2.16 versus 2.59 events per 100 person‐years; *P*=0.361) and all‐cause death (5.71 versus 6.39 events per 100 person‐years; *P*=0.361) were lower, but not significantly, in the low AIP group compared with the high AIP group (Table 2).

### Association of Thromboembolic Events With AIP Groups

**Figure 2:**
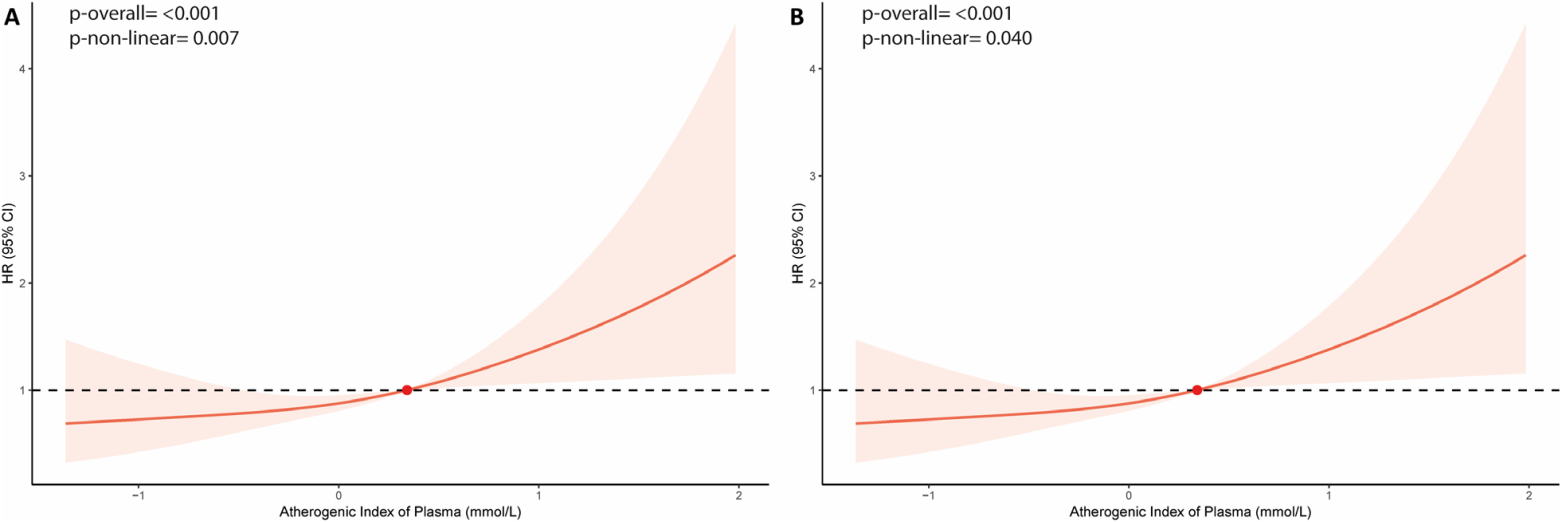
Restricted cubic spline analysis for thromboembolic events (A) and MACE (B). Adjusted for age, sex, AF type, hypertension, diabetes, prior stroke/TIA/thromboembolism, vascular disease, heart failure, chronic kidney disease, dyslipidemia, and smoking habit. AF indicates atrial fibrillation; HR, hazard ratio; MACE, major adverse cardiovascular events; and TIA, transient ischemic attack.

**Figure 3:**
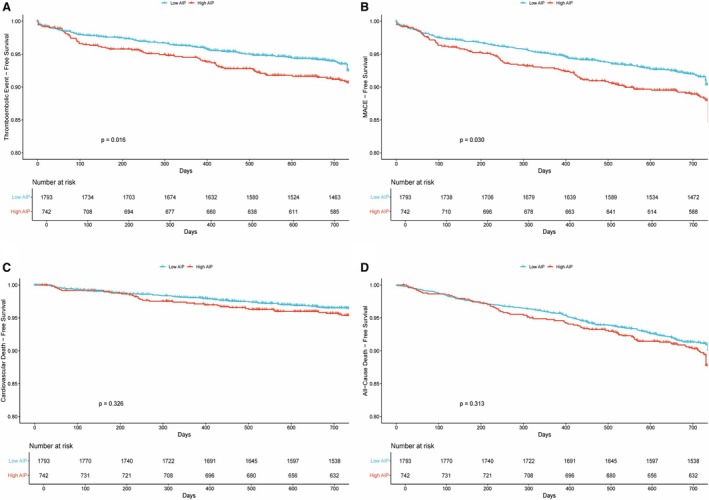
Kaplan‐Meier curves for outcomes in the AIP group. Panels **A–D** show (**A**) thromboembolic events, (**B**) MACE, (**C**) cardiovascular death, and (**D**) all‐cause death. Tick marks indicate censoring at the administrative end of follow‐up or at death (for nonfatal end points). AIP indicates Atherogenic Index of Plasma; and MACE, major adverse cardiovascular events.

Restricted cubic spline analysis demonstrated a significant overall (*P*‐overall <0.001) and nonlinear association (*P*‐nonlinear=0.007) between AIP and thromboembolic risk (Figure 2A). Kaplan–Meier analysis revealed significantly lower event‐free survival in the high AIP group compared with the low AIP group (log‐rank test, *P*=0.016) (Figure 3).

As shown in Table 3, patients in the high AIP group had a significantly higher risk of thromboembolic events than those in the low AIP group after adjustment for conventional cardiovascular risk factors and comorbidities (model 1: aHR, 1.47 [95% CI, 1.07–2.02]; *P*=0.018). This association remained statistically significant after further adjustment for commonly prescribed concomitant treatments (model 2: aHR, 1.38 [95% CI, 1.01–1.88]; *P*=0.040).

**Table 3:** HRs for the Different Outcomes According to the AIP Group

Variable	No. (%)	Model 1		Model 2	
		aHR (95% CI)	*P* value	aHR (95% CI)	*P* value
Any thromboembolic event					
Low AIP	120 (6.7)	Reference		Reference	
High AIP	67 (9)	1.47 (1.07–2.02)	0.018	1.38 (1.01–1.88)	0.040
MACE					
Low AIP	166 (9.3)	Reference		Reference	
High AIP	88 (11.9)	1.40 (1.07–1.84)	0.015	1.35 (1.03–1.76)	0.027
Cardiovascular death					
Low AIP	73 (4.1)	Reference		Reference	
High AIP	36 (4.9)	1.24 (0.82–1.86)	0.304	1.18 (0.78–1.79)	0.412
All‐cause death					
Low AIP	193 (10.8)	Reference		Reference	
High AIP	89 (12)	1.22 (0.94–1.59)	0.143	1.10 (0.85–1.43)	0.460

Model 1: adjusted for age, sex, atrial fibrillation type, hypertension, diabetes, prior stroke/transient ischemic attack/thromboembolism, vascular disease, heart failure, chronic kidney disease, dyslipidemia, and smoking habit.

Model 2: adjusted for oral anticoagulation, lipid‐lowering agents, oral hypoglycemic agents, insulin, and antiplatelet therapy.

aHR indicates adjusted HR; AIP, Atherogenic Index of Plasma; HR, hazard ratio; and MACE, major adverse cardiovascular events.

The cumulative incidence curves for thromboembolic events were consistent with these findings, showing a higher risk among patients with high AIP (Figure S1).

### Association of MACE With AIP Groups

Restricted cubic spline analysis showed a significant overall (*P*‐overall <0.001) and nonlinear association (*P*‐nonlinear=0.040) between AIP and MACE risk (Figure 2B). Kaplan–Meier analysis showed significantly lower event‐free survival in the high AIP group (log‐rank *P*=0.030) (Figure 3).

According to Table 3, patients in the high AIP group had a significantly higher risk of MACE than those in the low AIP group after adjustment for conventional cardiovascular risk factors and comorbidities (model 1: aHR, 1.40 [95% CI, 1.07–1.84]; *P*=0.015). This association remained statistically significant after further adjustment for commonly prescribed concomitant treatments (model 2: aHR, 1.35 [95% CI, 1.03–1.76]; *P*=0.027).

Similarly, cumulative incidence curves for MACE demonstrated a higher risk among patients in the high AIP group, in line with the primary results (Figure S1).

### AIP Groups and Secondary Outcomes

Kaplan–Meier curves revealed no significant differences between AIP groups for cardiovascular death (log‐rank *P*=0.330) or all‐cause death (log‐rank *P*=0.310) (Figure 3).

In multivariable Cox proportional hazards analyses, no statistically significant associations were observed between high AIP and either cardiovascular death or all‐cause death after adjustment for conventional cardiovascular risk factors and comorbidities (model 1: cardiovascular death: aHR, 1.24 [95% CI, 0.82–1.86]; *P*=0.304; all‐cause death: aHR, 1.22 [95% CI, 0.94–1.59]; *P*=0.143) or after further adjustment for commonly prescribed concomitant treatments (model 2: cardiovascular death: aHR, 1.18 [95% CI, 0.78–1.79]; *P*=0.412; all‐cause death: aHR, 1.10 [95% CI, 0.85–1.43]; *P*=0.460) (Table 3).

### Subgroup Analyses

**Figure 4:**
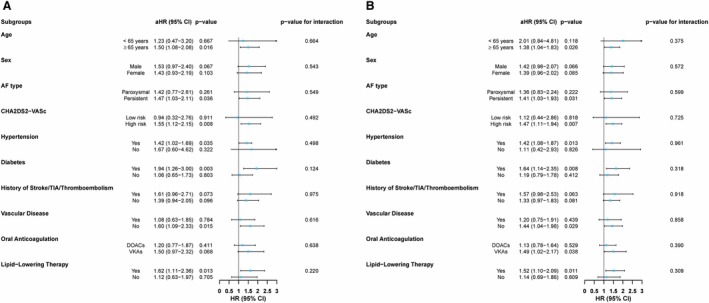
HRs for AIP across clinical subgroups for thromboembolic events (A) and MACE (B), derived from Cox regression analysis. Adjusted for age, sex, AF type, hypertension, diabetes, prior stroke/TIA/thromboembolism, vascular disease, heart failure, chronic kidney disease, dyslipidemia, and smoking habit. AF indicates atrial fibrillation; aHR, adjusted HR; CHA_2_DS_2_‐VASc, Congestive heart failure, Hypertension, Age ≥75 years (2 points), Diabetes, Stroke/transient ischemic attack/thromboembolism (2 points), Vascular disease, Age 65–74 years, Sex category; DOAC, direct‐acting oral anticoagulant; HR, hazard ratio; TIA, transient ischemic attack; and VKA, vitamin K antagonist.

After multivariable adjustment for conventional cardiovascular risk factors and comorbidities, the association between high AIP and the risk of thromboembolic events remained directionally consistent across all predefined clinical subgroups, with no statistically significant interactions observed (all *P* for interaction >0.05) (Figure 4A). Statistically significant associations with thromboembolic risk were observed in several subgroups, including patients aged ≥65 years (*P*=0.016), those with persistent AF (*P*=0.036), individuals with a high CHA_2_DS_2_‐VASc score (*P*=0.008), patients with hypertension (*P*=0.035) or diabetes (*P*=0.003), those without established vascular disease (*P*=0.015), and patients receiving lipid‐lowering agents (*P*=0.013).

Importantly, when stratified by type of OAC, the association between high AIP and thromboembolic events was directionally consistent in both DOAC‐ and VKA‐treated patients, with no evidence of effect modification by anticoagulant type (*P* for interaction=0.638) (Figure 4A).

Similarly, for MACE, high AIP was associated with an increased risk across most clinical subgroups after multivariable adjustment (all *P* for interaction >0.05), with statistically significant associations observed in patients aged ≥65 years (*P*=0.026), those with persistent AF (*P*=0.031), individuals with a high CHA_2_DS_2_‐VASc score (*P*=0.007), patients with hypertension (*P*=0.013) or diabetes (*P*=0.008), those without vascular disease (*P*=0.029), and those receiving lipid‐lowering agents (*P*=0.011) (Figure 4B).

Stratification by OAC type showed a directionally consistent association in both DOAC and VKA users, with no significant interaction between AIP and anticoagulant class (*P* for interaction=0.390).

### Discriminative Performance and Incremental Predictive Value of AIP for Thromboembolic Events

When the CHA_2_DS_2_‐VASc score was modeled as a categorical variable reflecting clinically used risk strata, receiver operating characteristic curve analyses showed a modest improvement in discriminative performance with the addition of AIP. The combined categorical model incorporating AIP demonstrated a higher area under the receiver operating characteristic curve compared with categorical CHA_2_DS_2_‐VASc alone (AUC, 0.612 versus 0.586; *P*=0.048) (Figure S2).

In reclassification, the addition of AIP was associated with a statistically significant integrated discrimination improvement (integrated discrimination improvement, 0.003 [95% CI, 0.001–0.006]; *P*=0.018). In contrast, categorical net reclassification improvement did not reach statistical significance (net reclassification improvement, −0.012 [95% CI, −0.068 to 0.043]; *P*=0.671).

## DISCUSSION

In this prospective cohort of anticoagulated patients with AF, several clinically relevant observations emerged: (1) Higher AIP was independently associated with an increased risk of thromboembolic events and MACE, even after adjustment for established clinical risk factors and commonly prescribed concomitant therapies. In line with this, patients in the high AIP category experienced shorter event‐free survival during follow‐up. (2) Cardiovascular death and all‐cause death rates were numerically higher in the high AIP group, but these differences did not reach statistical significance. (3) The associations between elevated AIP and both thromboembolic events and MACEs remained consistent across all predefined clinical subgroups after multivariable adjustment, with no evidence of meaningful effect modification, including when stratified by age, hypertension, diabetes status, CHA_2_DS_2_‐VASc risk categories, vascular disease, and type of oral anticoagulation. (4) Although the addition of AIP to the CHA_2_DS_2_‐VASc score resulted in only a modest improvement in discrimination when the score was applied using clinically relevant risk categories, these findings nonetheless support a complementary role for AIP in capturing aspects of underlying cardiometabolic risk and refining risk stratification beyond traditional clinical risk scores.

To our knowledge, this is the first prospective study to specifically evaluate the role of AIP in characterizing residual cardiovascular risk among anticoagulated patients with AF, a population in whom lipid‐related biomarkers may be particularly relevant given their prothrombotic milieu and frequent metabolic dysregulation.^24^ Previous studies have linked elevated AIP to cardiovascular and thromboembolic outcomes, but these investigations have largely focused on general populations or on patients with overt atherogenic conditions, such as diabetes,^9^, ^10^ coronary artery disease,^11^, ^12^ or metabolic syndrome,^25^ rather than on populations with AF receiving OAC therapy.

Despite adequate OAC, a substantial proportion of patients with AF continue to experience thromboembolic events,^6^, ^26^ underscoring the limitations of existing clinical risk scores and highlighting the contribution of non–thrombin‐related mechanisms to residual risk. Importantly, this residual risk should be interpreted within the context of an aging, multimorbid population with AF, in whom cardiometabolic dysregulation associated with multimorbidity may drive persistent thromboembolic and cardiovascular vulnerability.^27^, ^28^ In this setting, AIP appears to integrate multiple adverse metabolic signals into a single biomarker.

From a mechanistic perspective, the association between elevated AIP and adverse cardiovascular outcomes likely reflects a complex interplay of metabolic, inflammatory, and endothelial pathways.^29^ AIP captures the imbalance between elevated triglycerides and reduced HDL‐C, key features of atherogenic dyslipidemia.^7^ Hypertriglyceridemia promotes the formation of small, dense, low‐density lipoprotein cholesterol particles that are highly atherogenic, accelerating vascular inflammation and endothelial dysfunction,^30^ whereas reduced HDL‐C impairs reverse cholesterol transport and attenuates antioxidant and anti‐inflammatory vascular protection.^31^

These lipid abnormalities are closely linked to insulin resistance, visceral adiposity, and the metabolic syndrome,^25^ conditions that promote systemic inflammation and a prothrombotic state through oxidative stress, endothelial dysfunction, platelet activation, and increased levels of procoagulant mediators, such as plasminogen activator inhibitor‐1.^32^ In AF, already characterized by atrial remodeling, blood stasis, and chronic inflammation, this dyslipidemic and metabolic background may further amplify thrombotic susceptibility and contribute to cardiovascular risk that is not fully mitigated by anticoagulation alone.^33^ Moreover, elevated AIP may reflect subclinical atherosclerosis or microvascular dysfunction, mechanisms increasingly recognized as contributors to cardioembolic events in AF.

Despite the absence of direct comparisons, our findings are in line with existing literature. For instance, Qu et al^34^ examined the relationship between AIP and the incidence of new‐onset stroke in 8727 participants aged ≥45 years from the CHARLS (China Health and Retirement Longitudinal Study) cohort. Patients were divided into quartiles based on AIP, and compared with the first quartile, the higher quartiles were associated with a significant increase in the risk of new‐onset stroke (aHR, 1.34 [95% CI, 1.05–1.71]; aHR, 1.52 [95% CI, 1.19–1.93]; and aHR, 1.84 [95% CI, 1.45–2.34], for each successive quartile, respectively).

Similar results were reported by Zheng et al^14^ in a retrospective analysis of the Kailuan Study, which included 54 123 participants, where compared with the first AIP quartile, higher AIP quartiles were associated with a significantly elevated risk of ischemic stroke (aHR, 1.17 [95% CI, 1.03–1.32]; aHR, 1.33 [95% CI, 1.18–1.50]; and aHR, 1.45 [95% CI, 1.28–1.64], respectively).

In line with our findings, Liu et al^35^ investigated AIP in 3820 participants, and found that individuals in the highest quartile were more likely to experience MACE (aHR, 1.76 [95% CI, 1.27–2.44]) and stroke (aHR, 1.69 [95% CI, 1.17–2.45]); however, no significant association was observed between cumulative AIP and cardiovascular death.

Taken together, these data provide clinically relevant evidence that elevated AIP identifies a subgroup of anticoagulated patients with AF who remain at heightened residual cardiovascular risk. Although AIP does not replace established clinical risk scores, its independent association with thromboembolic events and MACE highlights the limitations of conventional stratification tools that do not account for metabolic and lipid‐related contributors. Rather, AIP may serve as a complementary biomarker to refine residual risk assessment and to characterize a high‐risk cardiometabolic AF phenotype despite adequate anticoagulation.

Because AIP is derived from routinely available triglyceride and HDL‐C measurements, its integration into clinical practice is feasible, inexpensive, and widely accessible. In this context, elevated AIP may support more individualized risk stratification and prompt closer follow‐up or more aggressive management of modifiable cardiometabolic risk factors, consistent with a precision‐medicine approach to AF care.

### Limitations

This study has several limitations that should be acknowledged. First, its observational design precludes definitive causal inference, and although we adjusted for a broad range of established confounders, residual confounding from unmeasured or unknown factors cannot be entirely excluded. Second, all participants were receiving oral anticoagulation at baseline, which limits the generalizability of our findings to anticoagulant‐naïve patients or those managed with nonpharmacologic strategies or alternative antithrombotic approaches. Third, the relatively high proportion of VKA use reflects real‐world anticoagulation practice in Spain during the study period, when regulatory and reimbursement constraints restricted de novo DOAC initiation despite their availability, potentially limiting the applicability of our findings to contemporary prescribing patterns.^15^ Although the median time in therapeutic range among VKA‐treated patients was ≈65%, indicating acceptable anticoagulation control, detailed longitudinal information on treatment adherence or temporal variability in anticoagulation quality was not available and may have influenced thromboembolic risk. Fourth, the study population was predominantly White and recruited from 2 academic centers in Spain, which may constrain the external validity of our results when extrapolated to more ethnically diverse or geographically distinct populations, particularly given known ethnic differences in the epidemiology of AF and AF‐related complications, such as stroke and bleeding. Fifth, lipid parameters, including triglycerides and HDL‐C, were measured only at baseline. Although this approach is standard in biomarker studies, it does not account for intraindividual variability over time or dynamic changes related to intercurrent illness, lifestyle modifications, or pharmacologic treatments. Emerging evidence suggests that longitudinal trajectories of lipid‐related biomarkers may provide more accurate prognostic information than single time‐point measurements, particularly for predicting MACE.^36^ Sixth, AIP was calculated using a logarithmic transformation of the triglyceride/HDL‐C ratio rather than through direct biochemical quantification. Although this method is widely accepted and validated, it may introduce some degree of measurement variability, particularly in individuals with borderline lipid values. Seventh, the binary cutoff used to define high AIP was derived in a data‐driven manner within the same cohort in which model performance was evaluated; consequently, the magnitude of the observed associations at this threshold may be overestimated, and external validation in independent cohorts is warranted. Eighth, event rates for certain outcomes, particularly cardiovascular death, were relatively low in some AIP subgroups, which may have limited statistical power and reduced the precision of effect estimates. Finally, the study did not evaluate other lipid‐related indexes or inflammatory biomarkers that could interact with AIP or provide complementary prognostic information.

## CONCLUSIONS

In this cohort of patients with AF on OAC, elevated AIP was independently associated with a higher risk of thromboembolic events and MACE, even after adjustment for conventional cardiovascular risk factors and commonly prescribed concomitant treatments. AIP may serve as a useful complementary marker of residual thromboembolic and cardiovascular risk in anticoagulated patients with AF.

## Sources of Funding

This work was supported by the Spanish Ministry of Economy, Industry, and Competitiveness, through the Instituto de Salud Carlos III after independent peer review (research grant: PI21/00670 cofunded by ERDF/ESF, “Investing in your future”); and by the Fundación Séneca–Agencia de Ciencia y Tecnología de la Región de Murcia (FSRM), through action 23042/GERM.

## Disclosures

José M. Rivera‐Caravaca: consultant for Idorsia Pharmaceuticals LTD. Francisco Marín: consultant and speaker for Boehringer‐Ingelheim and BMS/Pfizer. Gregory Y. H. Lip: consultant and speaker for BMS/Pfizer, Boehringer Ingelheim, Daiichi‐Sankyo, and Anthos. No fees are received personally. He is a National Institute for Health and Care Research Senior Investigator and co–principal investigator of the AFFIRMO project on multimorbidity in atrial fibrillation, which has received funding from the European Union's Horizon 2020 research and innovation program under grant agreement 899871. There is nothing to disclose for the other authors.

## Supporting information

Table S1
Figures S1–S2

STROBE Checklist
