# Supplementary material for: Atherogenic Index of Plasma and Residual Risk in Anticoagulated Patients With Atrial Fibrillation: The Prospective Murcia Atrial Fibrillation Project III Cohort
Source: J Am Heart Assoc. 2026 Mar 10;15(6):e046694. doi: 10.1161/JAHA.125.046694 (PMC13055707; doi:10.1161/JAHA.125.046694)
Supplement: Supplementary file 1 — Table S1 Figures S1–S2 [file JAH3-15-e046694-s001.pdf]

Supplemental Material

Table S1. Baseline clinical characteristics.

	**N = 2,535**
**Demographics**	
Age, median (IQR)	76 (69-82)
Sex [Female], n (%)	1,328 (52.4)
AF type, n (%)	
Persistent	1,611 (63.6)
Paroxysmal	924 (36.4)
**Comorbidities, n (%)**	
Hypertension	2,157 (85.1)
Diabetes mellitus	981 (38.7)
Heart failure	519 (20.5)
History of stroke/TIA/thromboembolism	595 (23.5)
Vascular disease*	542 (21.4)
Renal impairment	547 (21.6)
Dyslipidaemia	1,505 (59.4)
COPD/OSA	559 (22.1)
History of relevant bleeding	409 (16.1)
Liver disease	114 (4.5)
History of cancer	331 (13.1)
Smoking habit	613 (24.2)
Alcoholism	239 (9.4)
**Concomitant treatment, n (%)**	
Antiarrhythmics	428 (16.9)
ACE inhibitors	632 (24.9)
ARBs	1,146 (45.2)
Calcium channel blockers	748 (29.5)
Beta-blockers	1,702 (67.1)
Diuretics	1,476 (58.2)
Lipid-lowering agents	1,452 (57.3)
Oral hypoglycaemic agents	752 (29.7)
Insulin	223 (8.8)
Antiplatelet therapy	380 (15.0)
**Stroke and bleeding scores, median [IQR]**	
CHA_2DS_2-VA	4 (3-5)
CHA_2DS_2-VASc	3 (2-5)
HAS-BLED	3 (2-4)
**Type of OAC at baseline, n (%)**	
DOACs	1,725 (68.1)
VKAs	810 (31.9)

Abbreviations: IQR, interquartile range; TIA, transient ischaemic attack; COPD/OSA, chronic obstructive pulmonary disease/obstructive sleep apnoea; ACE, angiotensin-converting-enzyme inhibitors; ARBs, angiotensin II receptors blockers; DOACs, direct-acting oral anticoagulants; VKAs, vitamin K antagonists.

*Vascular disease includes coronary artery disease and/or peripheral artery disease.

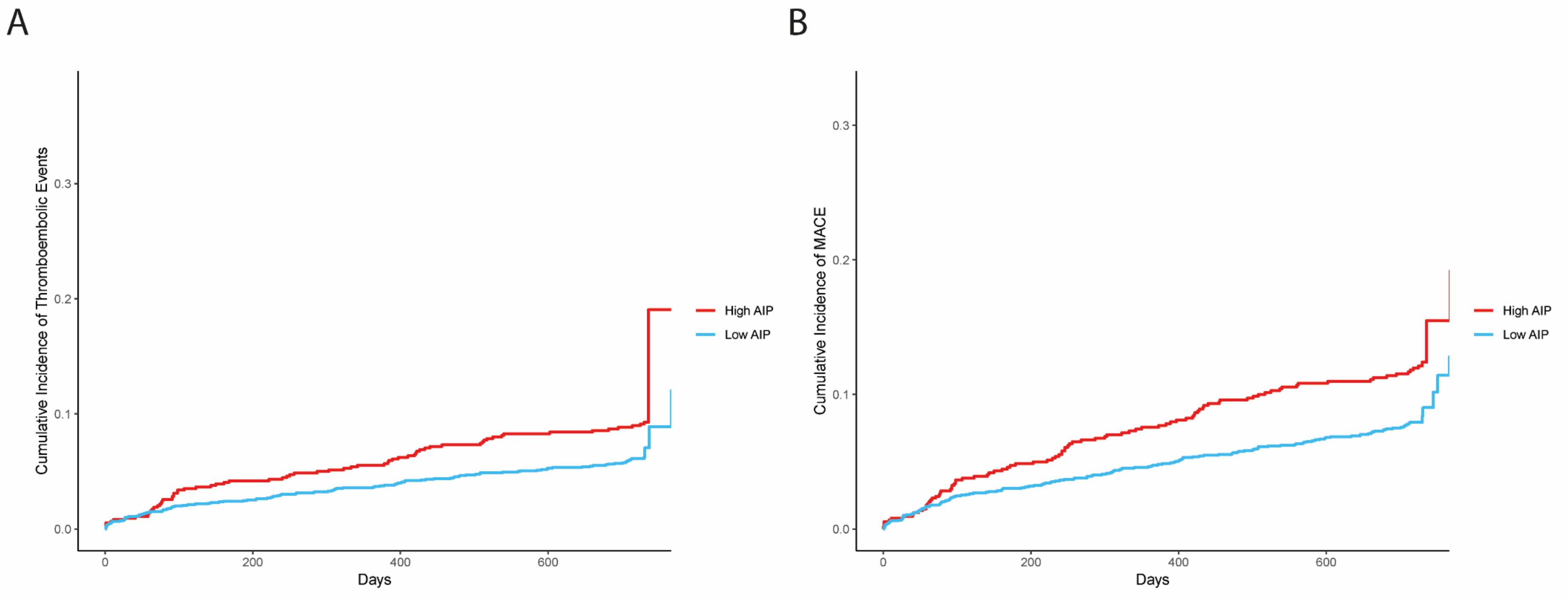

Figure S1. Cumulative incidence curves of thromboembolic events (a) and MACE (b) by AIP groups, accounting for the competing risk of death. AIP, atherogenic index of plasma.

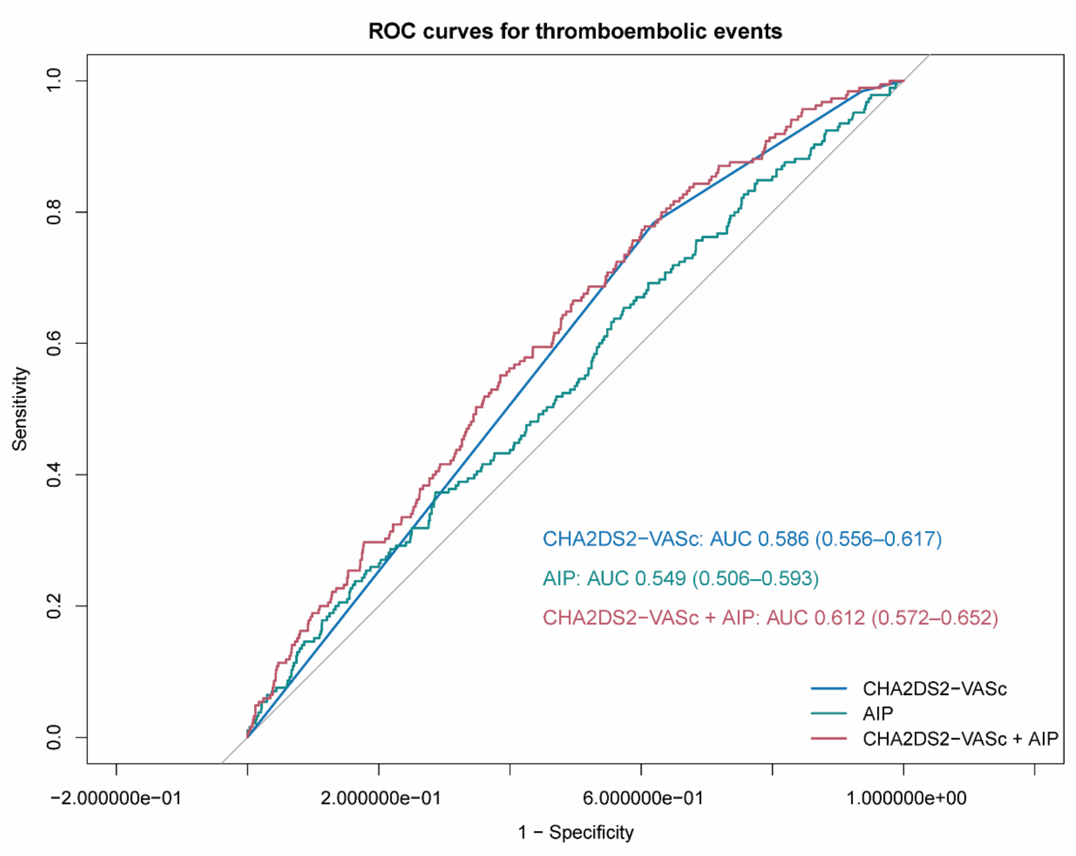

Figure S2. ROC curves for predicting thromboembolic events using AIP, categorical CHA_2DS_2-VASc, and their combination. AIP, atherogenic index of plasma.
